# Enhanced osseointegration of three-dimensional supramolecular bioactive interface through osteoporotic microenvironment regulation

**DOI:** 10.7150/thno.43736

**Published:** 2020-03-26

**Authors:** Haotian Bai, Yue Zhao, Chenyu Wang, Zhonghan Wang, Jincheng Wang, Hou Liu, Yubin Feng, Quan Lin, Zuhao Li, He Liu

**Affiliations:** 1 Orthopaedic Medical Center, The Second Hospital of Jilin University, Changchun 130041, P. R. China; 2 State Key Lab of Supramolecular Structure and Materials, College of Chemistry, Jilin University, Changchun 130012, P. R. China; 3 Orthopaedic Research Institute of Jilin Province, Changchun 130041, P. R. China; 4 Department of Plastic and Reconstruct Surgery, The First Bethune Hospital of Jilin University, Changchun 130021, P. R. China; 5 Department of Pain, Renji Hospital, South Campus, Shanghai Jiaotong University, Shanghai 201112, P. R. China

**Keywords:** bioactive interface, bone morphogenetic protein 2, osteoporotic microenvironment, osseointegration, supramolecular hydrogel

## Abstract

**Purpose**: Osteoporosis is more likely to cause serious complications after joint replacement, mainly due to physiological defects of endogenous osteogenic cells and the pathological osteoclast activity. It is a feasible solution to design a prosthetic surface interface that specifically addresses this troublesome situation.

**Methods**: A novel “three-dimensional (3D) inorganic-organic supramolecular bioactive interface” was constructed consisting of stiff 3D printing porous metal scaffold and soft multifunctional, self-healable, injectable, and biodegradable supramolecular polysaccharide hydrogel. Apart from mimicking the bone extracellular matrix, the bioactive interface could also encapsulate bioactive substances, namely bone marrow mesenchymal stem cells (BMSCs) and bone morphogenetic protein-2 (BMP-2). A series of *in vitro* characterizations, such as topography and mechanical characterization, *in vitro* release of BMP-2, biocompatibility analysis, and osteogenic induction of BMSCs were carried out. After that, the *in vivo* osseointegration effect of the bioactive interface was investigated in detail using an osteoporotic model.

**Results**: The administration of injectable supramolecular hydrogel into the inner pores of 3D printing porous metal scaffold could obviously change the morphology of BMSCs and facilitate its cell proliferation. Meanwhile, BMP-2 was capable of being sustained released from supramolecular hydrogel, and subsequently induced osteogenic differentiation of BMSCs and promoted the integration of the metal microspores-bone interface *in vitro and in vivo*. Moreover, the osteoporosis condition of bone around the bioactive interface was significantly ameliorated.

**Conclusion**: This study demonstrates that the 3D inorganic-organic supramolecular bioactive interface can serve as a novel artificial prosthesis interface for various osteogenesis-deficient patients, such as osteoporosis and rheumatoid arthritis.

## Introduction

Postmenopausal osteoporosis is a chronic bone disease characterized by alterations in the microstructure of bone and gradually compromised bone strength, predisposing patients to increased fracture risk 1. The osteoporotic microenvironment is deficient in bone marrow mesenchymal stem cells (BMSCs) and generally lacks osteogenic activity, leading to progressive bone loss and low osteogenic capacity 2, 3. The major clinical challenge faced during joint replacement in osteoporosis is severe complications, such as prosthesis interfacial displacement, loosening and even periprosthetic fracture 4, 5. Therefore, the construction of a novel bioactive prosthesis interface would be of high clinical significance to targetedly address these problems.

Titanium alloy, as the most commonly applied material of orthopedic implant, has a promising application prospect because of its high mechanical strength and corrosion resistance, but limited by its high stiffness, which consequently leads to stress- shielding induced osteolysis 6, 7. Three-dimensional (3D) printed porous titanium alloy (pTi) scaffolds can significantly reduce the stiffness, and be printed with the desired shapes and higher surface area, which have interconnected porosities for bone ingrowth 8-10. However, pTi implants may fail because of insufficient integration into the surrounding bone, given the smooth surface of titanium alloys and poor cellular adhesion for bone generation, especially under pathological conditions such as osteoporosis and rheumatoid arthritis 11, 12. In our previous work, injectable and self-healing supramolecular hydrogels were prepared by *in situ* crosslinking of N-carboxyethyl chitosan (N-chitosan) and adipic acid dihydrazide (ADH) with hyaluronic acid-aldehyde (HA-ALD). These soft materials have remarkable self-healing capacity and can retain their function and structures after external damage. This characteristic is beneficial in the maintenance of integrity of network structures and mechanical properties of bulk gels encapsulating sensitive biological drugs/cells and delivers them to target sites. Given these advantages, the administration of injectable supramolecular hydrogel into the inner pores of 3D printing porous metal scaffold can ameliorate the poor integration between 3D-printed porous titanium alloy and bone 13.

BMSCs, as vastly used tissue engineering cells, have shown significant functions in mitigating bone loss and improving bone microarchitecture in osteoporosis 14-18. In addition, the process of osseointegration is a series of complex biological events that relates to bone-forming cells as well as numerous osteogenic growth factors 19. Bone morphogenetic protein-2 (BMP-2) is one of the most widely used critical and potent osteogenic growth factors, with high efficiency in inducing osteogenic differentiation of BMSCs and promoting bone formation *in vivo* 20. To achieve an efficient bioactive interface osseointegration, the development of next-generation tissue engineering scaffolds, including optimized scaffolds, topical drug treatment (*e.g.*, growth factors), and cellular therapies (*e.g.*, stem cells), is a potential strategy 21.

**Scheme 1:**
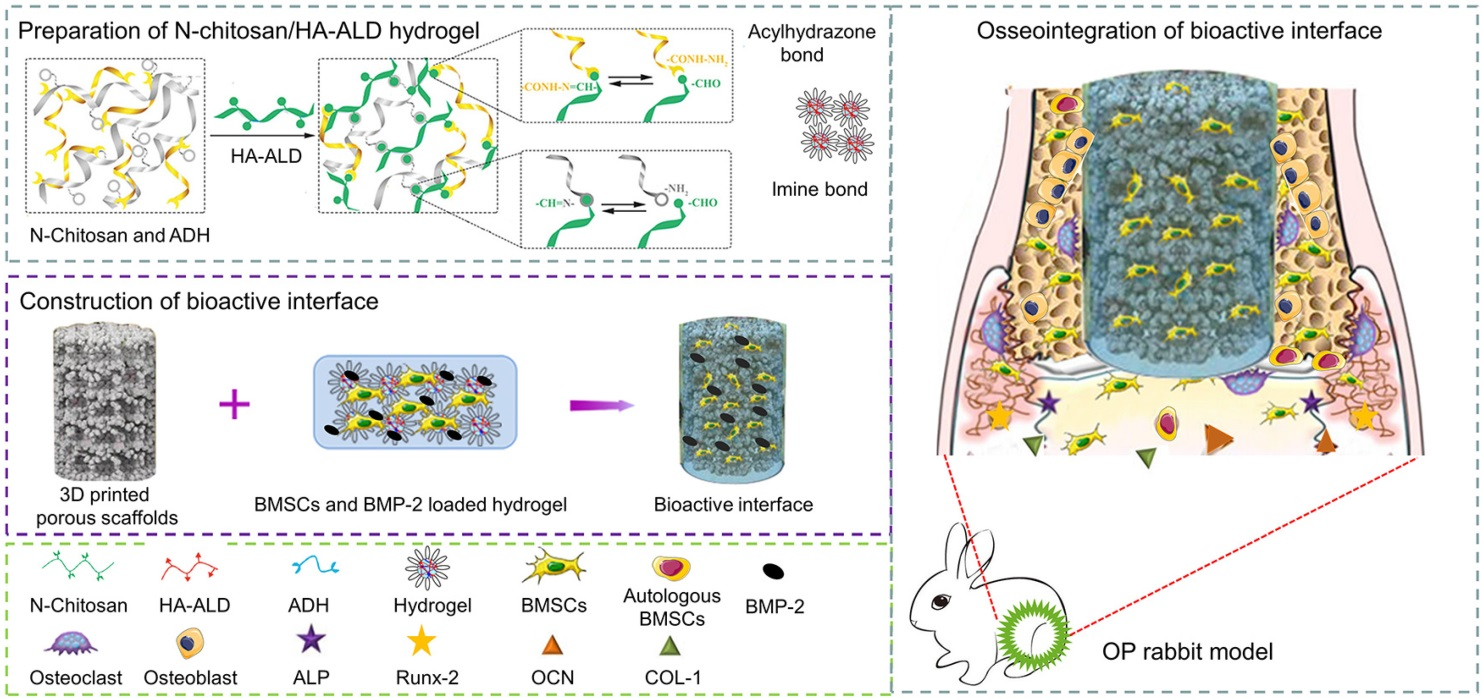
pTi filled with BMSCs and BMP-2 dual-loaded supramolecular hydrogels as bioactive composite scaffolds for enhancing osteoporotic bone defect osseointegration. BMP-2 can promote osteogenic differentiation of exogenous BMSCs and endogenous BMSCs. With the degradation of hydrogel, bone tissue grows into the pores of the pTi scaffold, thus achieving good osseointegration.

In this study, we developed a 3D inorganic-organic supramolecular bioactive interface simulating the dynamic network structure of bone matrix. The structurally and functionally optimized bioactive interface consists of printing porous metal scaffold and supramolecular polysaccharide hydrogel with BMSCs and BMP-2 dual-loading. The pTi implants have optimized porosity and pore size, which match the mechanical properties of the bone tissue to reduce stress shielding. Besides, the supramolecular polysaccharide hydrogel is self-healing, injectable, and biodegradable organic sacffold, which could encapsulate BMSCs and maintaining their vitality, as well as sustained release of bioactive BMP-2. The characterization, biocompatibility, and osteogenic differentiation ability of the bioactive interface were systematically investigated *in vitro*, and the osseointegration efficacy of the composite scaffolds was evaluated *in vivo* in a rabbit model of osteoporotic bone defect (**Scheme 1**). To the best of our knowledge, it is the first study to investigate the combination of stiff materials and supramolecular materials to fabricate 3D bioactive surface for BMP-2/BMSCs- mediated osteoporotic microenvironment regulation.

## Methods

### Materials

Spherical pre-alloyed medical-grade Ti6Al4V powder (Grade 23) was provided by AK Medical (Beijing, China). Chitosan (degree of deacetylation ~95%, viscosity 100-200 mPa.s) was supplied by Aladdin (Shanghai, China). Sodium periodate (NaIO_4_) and human BMP-2 enzyme-linked immunosorbent assay (ELISA) kit were got from Sigma-Aldrich (St. Louis, USA). Hyaluronic acid (100-200 k) and adipic acid dihydrazide (ADH) were supplied by Yuanye biology (Shanghai, China). Dulbecco's Modified Eagle's Medium (DMEM, low glucose), fetal bovine serum (FBS), and streptomycin-penicillin were purchased from Gibco^®^ Life Technologies (CA, USA). Medium for osteogenic differentiation of BMSCs was supplied by Cyagen Biosciences (Guangzhou, China). Cell Counting Kit 8 (CCK-8) and Calcein-AM/ Propidium Iodide (PI) were purchased from Dojindo Laboratories (Kumamoto, Japan). Rhodamine- phalloidin and 4',6-diamidino-2-phenylindole (DAPI) were obtained from Invitrogen (CA, USA). Eastep Super Total RNA Extraction Kit was provided by Promega (Shanghai, China), and Perfect Real Time RT reagent kit was supplied by Takara Bio (Dalian, China). Phosphate buffer (PBS) and 4% paraformaldehyde were purchased from Solarbio (Beijing, China). Hematoxylin, eosin, Masson, and Van-Gieson stains were purchased from Thermo Fisher Scientific (Shanghai, China). Triton X-100 was obtained from Dingguo Changsheng Biotechnology (Beijing, China). BMP-2, and antibodies used for immunofluorescence were purchased from Abcam (Cambridge, UK). Estrogen electrochemical immunoassay kit was purchased from Roche (Mannheim, Germany).

### Fabrication of porous Ti6Al4V scaffolds

Spherical pre-alloyed medical-grade Ti6Al4V powder (Grade 23, particle size 45-100 μm) was used for manufacturing the pTi alloy scaffolds by using additive manufacturing approach with an EBM system (Q10, Arcam, Sweden). In brief, the porous scaffolds were established on dodecahedron unit cells with the following design (nominal) dimensions: strut size = 300 mm, pore size = 800 μm, and porosity = 70%. Disk-shaped scaffolds (Ø10 mm × L3 mm) were used for microstructural and cellular biocompatibility and osteogenic assays *in vitro* (titanium plates without porosity were printed for the control group *in vitro* cell experiments), and the columnar-shaped scaffolds (Ø6 mm × L10 mm) were used for mechanical testing and *in vivo* osseointegration investigations. All samples were ultrasonically and sequentially cleaned in acetone, ethyl alcohol, and deionized water for ∼15 min for each treatment.

### Preparation of supramolecular polysaccharide hydrogel

The multifunctional supramolecular polysaccharide hydrogel was prepared as previously described 13. Concisely, hydrogel was prepared by *in situ* crosslinking of N-chitosan and ADH with HA-ALD; 7.5% N-chitosan (w/v) and 7.5% ADH (w/v) were dissolved in deionized water. Next, solutions of 5% HA-ALD (w/v) were added to the above mixture. For hydrogel formation by imine and acylhydrazone bonds, the solution was stirred using Lab Dancer to obtain a homogeneous hydrogel.

### Isolation and culture of rabbit BMSCs

BMSCs were extracted from a healthy one-week-old female New Zealand rabbit and cultured in DMEM supplemented with 10% FBS and 1% streptomycin-penicillin. The medium was changed every three days, and cell cultures were sustained in a humidified incubator at 37 °C and 5% CO_2_. When cells grew to ~80% confluence, the adherent cells were digested with 0.25% (w/v) trypsin/EDTA at 37 °C for 3 min and passaged, and the BMSCs were ready for further *in vitro* experiments after 3 passages.

### Bioactive interface construction

To prepare the 3D bioactive interface, BMSCs and BMP-2 were loaded into the N-chitosan and ADH solution, after which the HA-ALD was added to the above mixture. Referring to the protocol of the kit, a total of 2×10^5^ BMSCs and/or 50 μg BMP-2 was encapsulated into 600 μL N-chitosan and ADH solution with constant stirring. Next, 200 μL HA-ALD solution was added into the above solution, stirred gently, and injected into the pTi, which was incubated for 3 min to ensure full cross-linking of the hydrogels. Before gelation, BMSCs (final concentration, 2.5×10^5^ /mL) and/or BMP-2 (final concentration, 62.5 μg/mL) loaded hydrogels were injected into the pTi and placed inside a custom-made gel chamber. When it was fully gelled, the bioactive interface for experiments was constructed, and the components were shown in **Table S1**.

### Topography characterization of bioactive interface

The optical images of the hydrogel before and after gelatin formation were recorded with a digital camera (Canon EOS 550D, Japan). The sample porosity was measured according to the Archimedes' principle by the water-displacement technique. Porosity was further detected by microcomputed tomography (micro-CT) (voltage: 102 kV, current: 100 mA) (SkyScan 1076 scanner, Bruker micro-CT NV, Kontich, Belgium) and NRecon software (version 1.6.6; Bruker micro-CT). To quantify the average pore size and distribution, the sample microstructure was observed under a JEOL JSM-6700F scanning electron microscope (SEM), and images (*n* = 10) were analyzed by Image J software (National Institutes of Health, Bethesda, MD, USA).

### Mechanical characterization of bioactive interface

To detect the mechanical properties of the pTi, a universal testing machine (H25KS, Hounsfield, UK) was applied to measure uniaxial compressive strength of the scaffolds at a cross-head speed of 0.5 mm/min. Measurement of elastic modulus was performed using the microindentation technique by a micro indentation tester (MHT^3^; Anton Paar) fitted with Vickers diamond indenter (V-K 03), and a maximum load of 200 mN was used at an indentation rate of 400 mN/min with a maximum depth of penetration of 1000 μm. At least 10 measurements were carried out for testing each sample.

### *In vivo* degradation and biocompatibility analysis

To detect the degradation behavior and biocompatibility of the supramolecular hydrogel, *in vivo* degradation and biocompatibility examinations were carried out as described in our previous study 22. Briefly, 1 mL hydrogel was subcutaneously injected into the backs of Sprague-Dawley rats, and the rats were sacrificed at the predetermined time points (0, 7, 14, 21, and 28 d). Subcutaneously degraded gels were collected to take pictures with a digital camera and weighed. Hematoxylin and eosin (H&E) staining was performed to analysis the inflammation around the injection sites.

### Bioactive BMP-2 release profile

To investigate the release profiles of BMP-2 *in vitro*, the pTi scaffolds filled with BMP-2-loaded hydrogels were soaked in 2 mL DMEM supplemented with 10% FBS and 1% streptomycin-penicillin in a 24-well culture plate at 37 °C. At a predetermined time point, 2 mL medium was collected and stored at -80 °C, and another 2 mL fresh medium was added to the culture plate. The level of released BMP-2 in DMEM was analyzed by a BMP-2 ELISA Kit and a microplate reader (Multiskan EX, Thermo Fisher Scientific, Shanghai, China).

### Cell proliferation, viability and morphology

Nonporous Ti samples were sterilized and presoaked with complete DMEM for 24 h before using. In the CCK-8 experiment, the BMSCs were seeded on the nonporous Ti as control (Con), seeded in the porous pTi scaffold (S), incorporated into hydrogel to fill pTi scaffold (SG), or incorporated with BMP-2 into hydrogel to fill pTi scaffold (SGB) at a density of 5 × 10^4^ cells/well in 48-well culture plates, respectively. In addition, the proliferation of BMSCs seeded on the surface of scaffold (not inside) in Con, S, SG, and SGB groups was also studied. Then, the culture sustained for 1, 7, and 14 d to assess the cell proliferation by using CCK-8.

Due to the difficulty of fluorescent staining, BMSCs at a density of 5 × 10^4^ cells per sample were seeded on the surface of scaffold to observe cell viability and morphology in Con, S, SG, and SGB groups. Cell viability on samples after seeding with BMSCs was assessed using a Calcein-AM/PI Double Stain Kit according to the manufacturer's protocol after the culture for 3 d, and evaluated with a confocal laser scanning microscope (CLSM, FV1000, Olympus, Japan). For morphological assessment, the samples after 7 d of cell culture were fixed with 4% paraformaldehyde, then permeabilized with 0.1% solution of Triton X-100, and washed with PBS (pH = 7.4) repeatedly followed by rhodamine-phalloidin and DAPI staining per the manufacturer's protocol. The images of stained samples were captured under a fluorescent microscope.

### Induced osteogenic differentiation of BMSCs *in vitro*

To study the osteogenic ability of BMSCs induced by bioactive interface, BMSCs were seeded in Con, S, SG, and SGB groups at a density of 1×10^6^ cells per sample and cultured in 1 mL DMEM supplemented with 10% FBS and 1% streptomycin- penicillin. After 24 h, samples were complemented with osteogenic medium including low-glucose DMEM along with β-glycerol-phosphate (10 mM), ascorbate-2-phosphate (50 μM), and dexamethasone (0.1 μM). The medium was changed every 3 d. After osteogenic induction culture for 7 and 14 d, alizarin red staining (ARS) was performed to evaluate osteogenic differentiation.

In addition, after treatment with osteogenic medium for 7 and 14 d, the expressions of alkaline phosphatases (*ALP*), runt-related transcription factor-2 (*Runx-2*), osteocalcin (*OCN*), and collagen type-1 (*Col-1*) was detected by real-time quantitative PCR (RT-qPCR) to assess the differentiation of the cultured cells. The sequences of primers were listed in **Table S2**. After osteogenic culture for 14 d, immunocytochemical staining was also conducted per the manufacturer's instructions to study the expressions of *ALP*, *RUNX-2*, *OCN*, and *COL-1*. DAPI staining was used to mark the nucleus, and the samples were then imaged with CLSM. After that, the fluorescence intensity of these osteogenic related proteins in each group was quantitatively analyzed by Image J software.

### Preparation of osteoporotic bone defect models

All animal experimental protocols were approved by the Animal Care and Use Ethics Committee of The Second Hospital of Jilin University. The osteoporotic rabbit models were prepared by bilateral ovariotomy (OVX). Briefly, a total of 36 female New Zealand white rabbits, eight-month-old, were randomized into 2 groups to receive different surgeries: Group 1 (*n* = 33) rabbits underwent bilateral OVX surgery, and group 2 (*n* = 3) rabbits were subjected to sham surgery, namely close the abdominal cavity after finding the ovaries without any treatment. Each rabbit was kept in a cage individually and fed with standard chow. Serum estrogen levels of different groups were evaluated by an electrochemical immunoassay kit at 8 months after the surgery. Simultaneously, 3 animals from each group were sacrificed, and the bone tissues from distal femurs were harvested for H&E staining and micro-CT measurement to confirm the osteoporosis status.

### Implantation of bioactive interface

Residual 30 osteoporotic rabbits at 8 months post-OVX were included in the *in vivo* osseointegration experiments, which were randomly divided into five groups of six animals each: (I) pTi scaffold (S), (II) pTi scaffold filled with hydrogel (SG), (III) pTi scaffold filled with cell-loaded hydrogel (SGC), (IV) pTi filled with BMP-2-loaded hydrogel (SGB), and (IV) pTi filled with cells and BMP-2-loaded hydrogel (SGCB). In brief, the rabbit was anesthetized by 3% (w/v) pentobarbital with 50 mg kg^-1^. After skin preparation and sterilization, a longitudinal incision was made in the distal femur of the left hind limb for exposing the lateral condyle. The lateral condyle was drilled to induce a cylindrical bone defect (6.0 mm diameter and 10.0 mm depth), and according scaffolds were then transplanted into the defects after the drill-hole defect was washed using saline followed by hydrogen peroxide. The incisions were closed in layers by absorbable sutures. Postoperatively, the rabbits were allowed free movement, and penicillin (40,000 U) was injected intramuscularly for 3 d after surgery to prevent infection. Three months after scaffold implantation, all animals were sacrificed and their bilateral femurs were collected for micro-CT, histology, and push-out tests to explore the bone ingrowth and osseointegration of different scaffolds in osteoporotic bone defects.

### Micro-CT analysis

To study the effect of bone growth into the scaffolds, the specimens were subjected to micro-CT examination. The region of interest (ROI) was selected as a cylinder (diameter: 6 mm and height: 10 mm) centered on the scaffolds, and the 3D images were reconstructed. To estimate the quality of bone ingrowth, quantitative morphometric analysis of the ROI was carried out by micro-CT auxiliary software (NRecon version 1.6.6) to reconstruct and extract the related parameters, such as bone volume/tissue volume (BV/TV) ratio, trabecular thickness (Tb.Th, mm), trabecular separation (Tb.Sp, mm), and trabecular number (Tb.N, 1/mm).

### Histological examination

After micro-CT analysis, these samples were used for histological examination. The samples of bone tissue and scaffold complexes were dehydrated in a series of graded ethanol (70%, 80%, 95%, and 100%) for 2 h and then embedded in methyl methacrylate. Thin slices with 150-300 μm thickness were cut from the samples and ground to a thickness of 40-50 μm through the transverse saw cuts and polishing machine (Exact band saw; Exact Apparatebau, Norderstedt, Germany). After the preparation of hard tissue sections, the samples were stained with 1.2% trinitrophenol and 1% acid fuchsin, namely Van-Gieson staining.

In addition, bilateral proximal femurs were immersed in 10% ethylene diamine tetraacetic acid to demineralize for 1 month, after which the specimens were embedded in paraffin and sectioned into 5 μm slices by the microtome. Then, prepared sections were stained with H&E and Masson's Trichrome to investigate the effects of composition implants on the ipsilateral and contralateral proximal femur.

### Push-out tests

A standard push-out test for the scaffolds (*n* = 3, each group) was used for shear strength evaluation of the bone-implant interface. The specimens were displaced on the plate for the detaching test at 0.1 mm/s using a closed-loop servo-hydraulic testing machine (MTS MiniBionix, Minneapolis, USA). The load at which the scaffolds became detached from the bone was recorded as the maximum load.

### Statistical analysis

All data were expressed as means ± standard deviation (SD), and the statistical analysis were carried out using ANOVA with Tukey's post-hoc analysis (SPSS Inc., Chicago, IL, USA). P < 0.05 was considered to indicate statistical significance. All experiments were performed at least in triplicate.

## Results and Discussion

### Characterizations of bioactive interface

**Figure 1:**
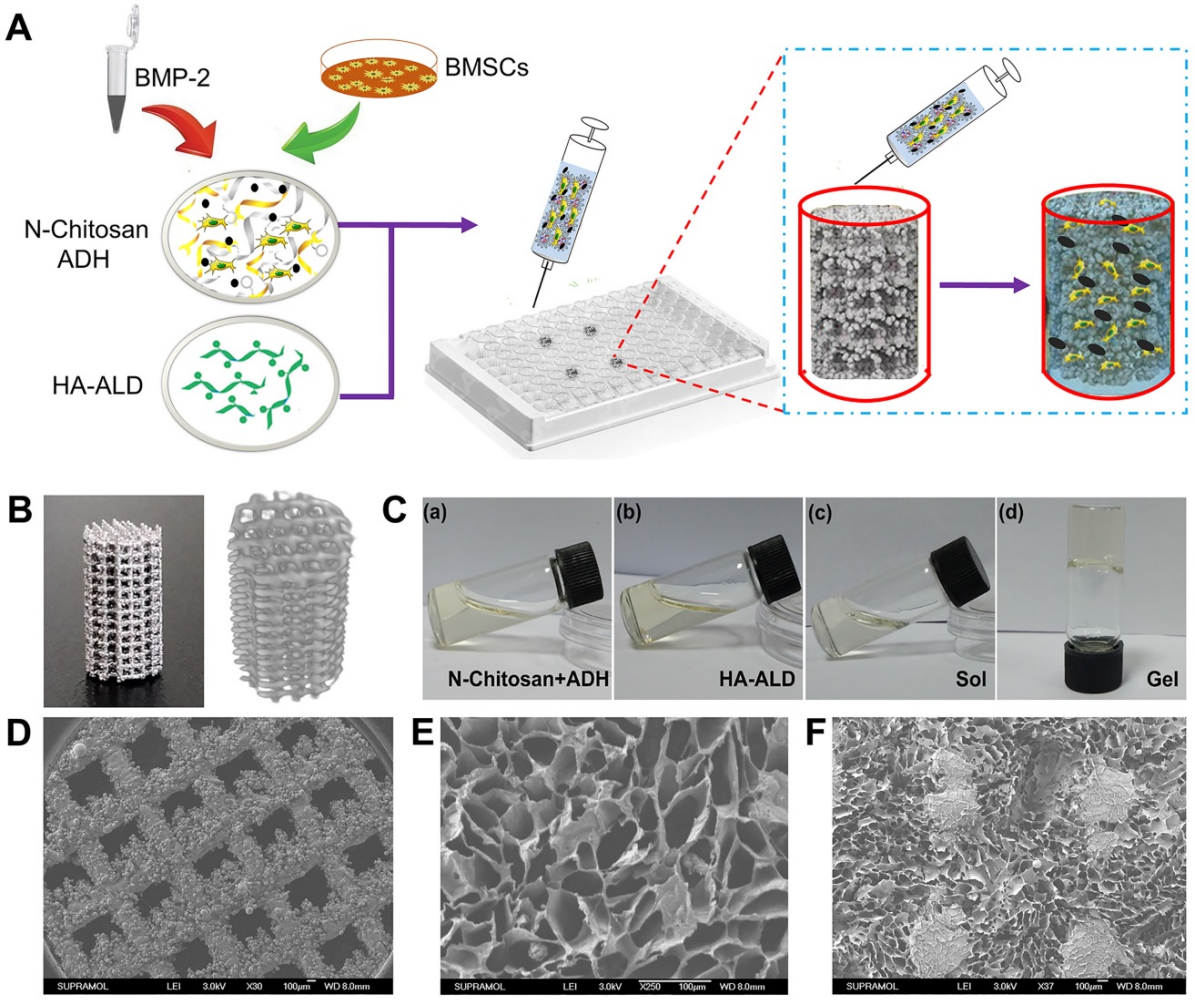
(**A**) Bioactive interface constructed by BMSCs and BMP-2 loaded hydrogel filled the pTi in a cell culture plate; (**B**) Representative optical image and 3D micro-CT image of pTi; (**C**) Mixed hydrogel and gelation; SEM images of (**D**) pTi, (**E**) supramolecular hydrogel, and (**F**) pTi filled with hydrogel.

As shown in **Figure 1A**, to prepare the 3D bioactive interface, the pTi was placed inside a cell culture plate as the custom-made gel chamber. BMSCs and BMP-2 dual-loaded hydrogel could be injected into the chamber through a syringe to fill the pores of pTi. The physical and chemical characteristics of the composite scaffolds were studied in detail. The porosity parameters of the pTi were carried out by Archimedes' principle and micro-CT, and pore size distribution of pTi was observed by SEM. The porosity of the pTi was in line with the original design (70%). Specifically, the detection of porosity through Archimedes' principle demonstrated that the porosity of pTi was 70.5±0.9%. Furthermore, the porosity percentage by micro-CT assessment was similar at 69.2±0.9%. Representative optical images and 3D micro-CT pictures of the pTi scaffolds were displayed in **Figure 1B**. Meanwhile, the sol-to-gel phase transformation process was visualized in **Figure 1C**, whose mechanism was attributed to acylhydrazone and imine bonds. SEM photographs of the pTi samples were also showed in **Figure 1D**, and then Image J analysis of the SEM pictures revealed the pore size of the pTi scaffolds to be 793.4±16.9 μm. The osseointegrative capacity of scaffolds usually depends on the osteoconductivity of the scaffold material, which further related to the extent of porosity, pore size and distribution of the scaffolds. The porosity of cancellous bone is generally 75-90%, and the diameter of the connected trabecula is usually about 50-300 μm 23. For bone tissue engineering porous scaffolds, porosity should be above 50%, especially within the scope of 60-70%, structurally and mechanically similar to human trabecular bone. While, a 50-500 μm pore size is favorable for osteoblast adhesion, proliferation and differentiation 10, 24. Therefore, the porosity and pore size of pTi scaffold in this study were suitable for bone tissue engineering. Increasing the surface area and porosity of the scaffolds could improve the initial implant stability, bone ingrowth capacity, and the friction coefficient between the bone and scaffolds, thus reducing micromotion and promoting osseointegration after implanting *in vivo* 25. Meanwhile, this relatively large pore was designed to reduce the elastic modulus of the metal scaffold and facilitate the filling of bioactive hydrogel, and the hydrogel with small pore size could in turn reduce the macropores of metal scaffolds.

The load versus displacement curve during compressive strength measurement was converted into stress versus strain curve, and the corresponding uniaxial compressive strength was estimated from the curves. The values of mechanical strength were presented in **Table S3**. Briefly, the compressive strength and elastic modulus of pTi were 48.0±2.1 MPa and 1.63±0.2 GPa, respectively. Considering the mechanical properties of scaffolds, cortical bone exhibits compressive strength and elastic modulus in the range of 100-250 MPa and 7-20 GPa, respectively; the respective values for cancellous bone range from 11 to 24 MPa and from 1.5 to 11.2 GPa, respectively 26. The compressive strength and elastic modulus of the 3D-printed pTi were obviously decreased compared with those of titanium and its alloys, which could effectively avoid stress-shielding and osteolysis 27.

As presented in **Figure 1E-F**, the morphology of the hydrogel, with or without pTi, were characterized by SEM. The pTi has large pore size, which may be unfavorable for cell attachment, proliferation, and intercellular communication. After modification with hydrogels, the large pores of pTi were filled with hydrated 3D porous structure similarly to native extracellular matrix. After lyophilization, the hydrogel was demonstrated possessing a homogenous porous structure with pore diameters ranging from 100 to 200 μm. When BMSCs were encapsulated into the hydrogel, the nanochannels of the hydrogel were beneficial to oxygen and nutrient exchange, thereby enhancing cell growth and communication, and further promoting bone regeneration 28.

In addition, the host response of the hydrogel is another critical issue for the implantation materials. Although slight inflammatory reaction was observed after subcutaneous injection for 0, 7, and 14 d (blue arrow), it gradually turned to be normal along with the degradation indicating that the hydrogel was acceptable for implantation with good biocompatibility. Because the subcutaneous hydrogel was stripped from the skin and weighed to calculate the degradation rate, only a small amount of dispersed hydrogel fragments (red arrows) could be seen in the dermis (**Figure S1**). Because the biomaterials must fill a defect and gradually degrade to allow for tissue ingrowth, hence, the control of degradation rates is considerably important. The hydrogel used in this research could completely degraded *in vivo* within 28 d (**Figure S2**). A previous study reported that angiogenesis and osteogenesis occurred in about 2-4 weeks after implantation of a BMP-2-loaded hydrogel 29. Herein, our hydrogel completely degraded *in vivo* within 4 weeks without causing significant inflammation, which not only matched the rate of bone ingrowth but also provided space for bone ingrowth into the pTi.

### Release profile of BMP-2

Previous studies have shown that proper concentration of BMP-2 (10-50 μg/sample) is beneficial to local bone regeneration and biological safety for the rabbit model of local bone defects 30-32. In consideration of the sustained-release manner of the bioactive interface used in our study, the dosage of BMP-2 was designed as 50 μg/sample. The release profile of BMP-2 from the bioactive interface was conducted *in vitro* (**Figure S3**). BMP-2 displayed slightly fast initial release with no initial burst release during the first day, and the cumulative half dose (48.7±2.1%) occurred in the 7th d. The release kinetics became slow after 5 d and maintained at a steady rate until 42 d. The suppressing burst release and maintaining sustained release of BMP-2 from the hydrogel system are very beneficial in promoting bone regeneration 29. This phenomenon was owing to the BMP-2-loaded hydrogel with self-healing or self-repairing characteristics, which could retain its functionality and structure after damage, which was advantageous in the maintenance of integrity of network structures and mechanical properties of bulk gels.

### Cell viability, proliferation and morphology

**Figure 2:**
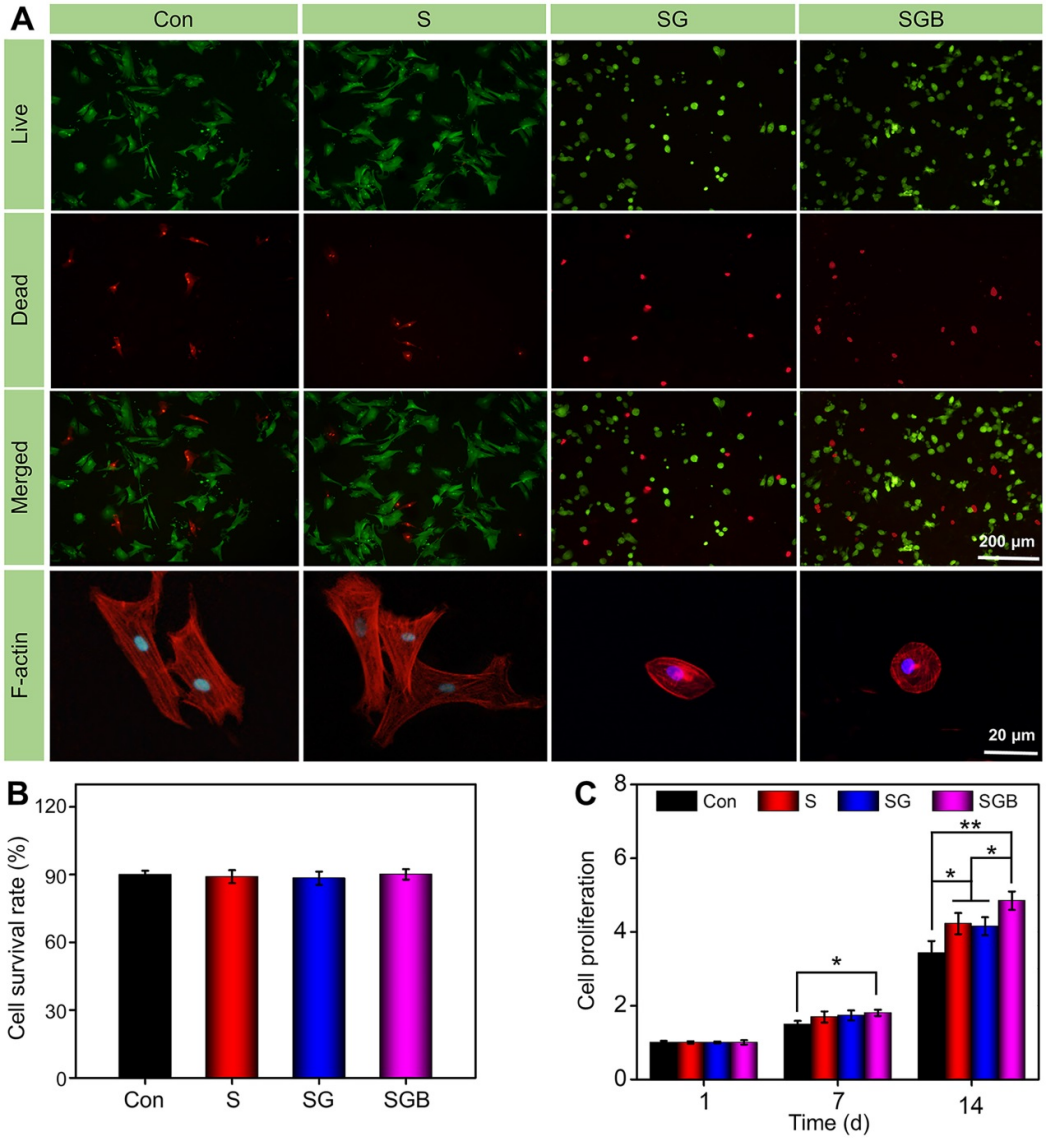
(**A**) Calcein AM/PI staining of live cells (green) and dead cells (red), and fluorescent imaging with rhodamine-DAPI staining of Con, S, SG, and SGB groups; (**B**) Quantitative analysis of cell survival rate by Calcein AM/PI staining; (**C**) Cell proliferation within the different scaffolds at 1, 7, and 14 d (*p < 0.05, **p < 0.01).

BMSCs seeded in different manners were stained with Calcein AM/PI to assess the cell viability. As shown in **Figure 2A**, the fluorescence microscope images clearly showed that most BMSCs could maintain good cell viability in different pTi scaffolds and nonporous scaffolds. In addition, the survival rate of BMSCs was calculated by Image J software. Quantitative analysis showed that the survival rates in the Con, S, SG, and SGB groups were 90.03±1.7%, 89.0±2.9%, 88.4±2.9%, and 90.1±2.3%, respectively, and there was no significant difference between these groups (**Figure 2B**). This result meant that titanium alloy scaffold and polysaccharide hydrogel had good biocompatibility.

Cell proliferation was detected by the CCK-8 assay in various samples. As shown in **Figure 2C**, for loading the BMSCs within the hydrogel, the cell proliferation was significantly greater in the SGB group than in the Con group at 7 d (p < 0.05). At 14 d, the SGB group not only had faster proliferation compared with the Con group (p < 0.01), but also showed significant difference compared with S and SG groups (p < 0.05). The similar result was obtained for loading BMSCs on the surface of bioactive interface (**Figure S4**). BMP-2 has been demonstrated to be an active inducer of BMSCs proliferation and differentiation 33. These results indicated that BMP-2-loaded hydrogel promoted the proliferation of BMSCs. On the other hand, compared with the Con group, the pTi scaffolds with porous structure showed a positive trend towards cell proliferation; and these was more significant in the pTi scaffolds with hydrogel attributed to the 3D cell culture system of hydrogels facilitating cell proliferation and adhesion 34.

Furthermore, cellular F-actin was stained with rhodamine-phalloidin and examined by fluorescence microscopy to investigate the cell morphology, when BMSCs were seeded on different scaffolds. BMSCs cultured on pTi scaffolds showed better spreading than those on nonporous Ti scaffolds and displayed more lamellipodia extensions, suggesting that the pTi scaffolds could induce cell migration 35. BMP-2 released from hydrogel in the SGB group promoted pronounced cytoskeletal reorganizations and F-actin augmentation, compared to the SG group with lacked BMP-2 loading. It is well-known that the F-actin filament plays a key role in the early maturation of BMSCs, and contributes to enhance bone cell functions by targeting cells proliferation and differentiation 36. Therefore, the BMP-2 loaded hydrogel can significantly enhance the fluorescence intensity of F-actin filaments and cytoskeletal organization, which is beneficial to the early osteogenic differentiation of BMSCs.

### Effect on osteogenic differentiation of BMSCs

In addition to cell adhesion and proliferation, cell differentiation is essential for bone regeneration. BMPs family is potent osteoinductive growth factor that induce ectopic bone formation 37. Of these, BMP-2 is one of the most potent osteoinductive cytokines and has been demonstrated to initiate the differentiation of BMSCs into osteoblasts in several animal models 38. The mineralized matrix synthesis and transcriptional level of osteogenic-related genes were explored by ARS staining assay and quantitative RT-qPCR to evaluate BMSCs differentiation, respectively.

**Figure 3:**
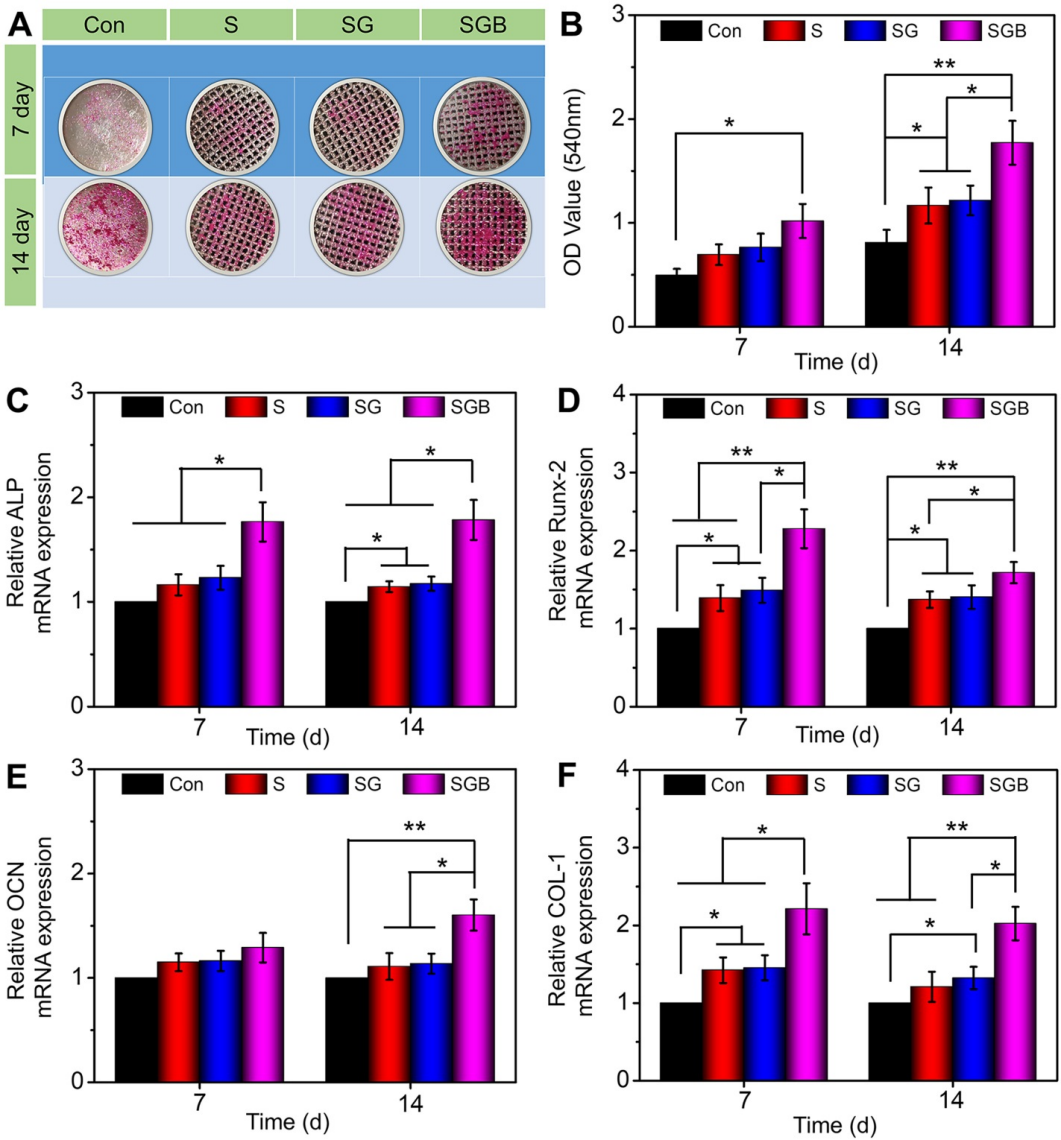
Evaluation of the osteogenic differentiation of BMSCs seeding in the Con, S, SG, and SGB groups. (**A**) Images of different samples after staining with alizarin red S; (**B**) Statistical analysis of semi-quantitative analysis of alizarin red staining; (**C-F**) The expressions of osteogenic-related genes, such as *ALP, RUNX-2, OCN, and COL-1* (*p < 0.05, **p < 0.01).

Gross images showed that the spots of calcium deposits in the Con group were limited at 7 d. In SGB group, visible calcium deposition was observed, which was more significant for 14 d (**Figure 3A**). The semi-quantitative analysis of ARS results was further indicated in** Figure 3B**. A significantly greater amount of calcium deposition was observed in the SGB group than in the Con, S, and SG groups (p < 0.01). Remarkably, with regard to porous structure of pTi and hydrogel-filled pTi, cells in the S and SG group exhibited significantly greater calcium deposition than those in the Con group (p < 0.05). These findings demonstrated that the present pTi and hydrogel promoted the formation of mineralized matrix and, further, increased the efficiency of mineralization after incorporating BMP-2.

For further quantitatively analysis the effect of composite scaffolds on osteogenic differentiation of BMSCs, the expressions of osteogenic-related genes were detected by RT-qPCR, namely *ALP, RUNX-2, OCN,* and* COL-1* (**Figure 3C-F**). Typically, *ALP* activity is measured to identify the osteogenic differentiation, and the upregulation of *ALP* activity is a key event happening in early osteogenesis 39. At both 7 and 14 d, the SGB group exhibited significantly higher expression of *ALP* than the Con, S, and SG groups (p < 0.05). Furthermore, with the prolongation of osteogenic induction, the expression of *ALP* in pTi scaffolds was also slightly higher (p < 0.05) than in nonporous Ti (**Figure 3C**). *Runx-2* is a member of the Runx family of transcription factors and is also one of the earliest indications of osteoblastic differentiation. *Runx-2* can induce the expression of key osteogenic genes such as *OCN* and osteopontin (*OPN*) 40, 41. The expression level of *Runx-2* was shown in **Figure 3D**, where obvious differences have already occurred on day 7. The expression of *Runx-2* in SGB group was significantly higher than Con and S groups (p < 0.01), and SG group (p < 0.05); and the expressions of *Runx-2* in S and SG groups were higher than that of Con group for 7 d. And these statistical differences were still significant for 14 d. *OCN* is a kind of bone-specific protein synthesized by osteoblasts and is recognized as a marker to assess osteogenic maturation and bone formation, and it shows the highest level during the late stage of osteogenesis 42, 43. **Figure 3E** revealed that the levels of *OCN* were not significantly different among different groups at the early stage, namely day 7; however, on day 14, compared with the Con, S, and SG groups, the SGB group showed enhanced levels of *OCN* expression by 1.5-fold, 1.4-fold, and 1.4-fold (all p < 0.05), respectively. In addition, *COL-1* is another critical osteogenic differentiation gene, and **Figure 3F** indicated that the expression level of *COL-1* in SGB group was significantly higher than those in Con, G, and SG groups for 7 and 14 d, which showed similar trend with *ALP, RUNX-2* and* OCN*.

**Figure 4:**
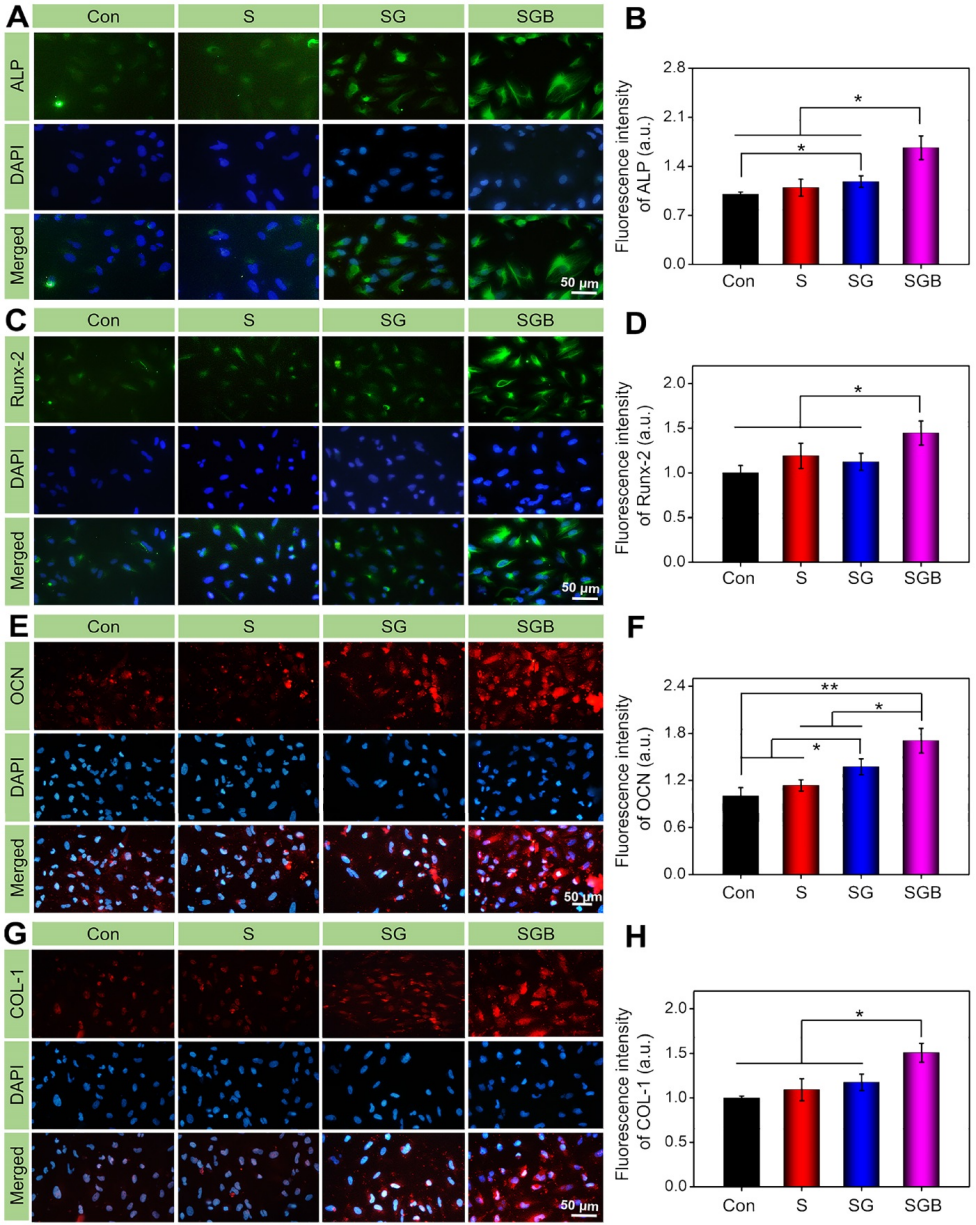
Immunohistochemical staining and quantitative analysis of osteogenic related proteins in BMSCs cultured in different groups for 14 d, namely (**A**, **B**) *ALP*, (**C**, **D**) *Runx-2*, (**E**, **F**) *OCN*, and (**G**, **H**) *COL-1* (*p < 0.05, **p < 0.01).

In order to further verify the effect of BMP-2 loaded hydrogel on osteogenic differentiation of BMSCs at protein level, the expression of osteogenic proteins (*ALP*, *RUNX-2*, *OCN*, and *COL-1*) were investigated by immunofluorescence staining. According to **Figure 4A**, BMSCs cultured on the SGB group had stronger fluorescence intensity compared to the Con, S, and SG groups, indicating that there was more *ALP* protein expression. Quantitatively, the expression of *ALP* in BMSCs cultured on the SGB was calculated as 1.67, 1.53 and 1.40 times higher than that of the Con, S, and SG groups (p < 0.05), respectively. Meanwhile, the fluorescence intensity of *ALP* in SG group was also significantly higher than that in the Con group (**Figure 4B**). For *RUNX-2*, the SGB group showed higher protein expression than the Con, S, and SG groups (p < 0.05, **Figure 4C-D**). In addition, the SG group was observed with significantly enhanced fluorescence intensity of *OCN* compared with that of the Con group (p < 0.01), and S and SG groups (p < 0.05). And the expression of *OCN* in the SG group was also higher than those in the Con and S groups (p < 0.05, **Figure 4E-F**). As shown in **Figure 4G-H**, the expression of *COL-1* in SGB group was up-regulated by 1.51, 1.38 and 1.29 times than those of the Con, S, and SG groups (p < 0.05), respectively. These results of *in vitro* study revealed that the BMP-2 loaded scaffolds played an essential role in promoting BMSCs osteogenic differentiation.

### Characterization of OVX rabbit models

All the animals sustained alive during the experimental process, and no infectious sign or other surgical complications were observed. Six animals were sacrificed to verify the success of osteoporotic model, which were randomly selected from the OVX (*n* = 3) and sham (*n* = 3) groups at 8 months after bilateral OVX. Serum estrogen level significantly descended in the OVX group compared to the sham group (p < 0.01) as shown in **Figure S5**. In the OVX group, the trabecular bone structure became looser and thinner, and more adipocytes inside the bone marrow implied the replacement of red marrow by adipose-rich yellow marrow as a result of estrogen depletion (**Figure S6**, left) 44. The CT images were shown in **Figure S6** (right), and statistical analyses showed that the bone mineral density (BMD) and BV/TV in the OVX animals were significantly lower than those in the sham group (**Figure S7A-B**). These results collectively demonstrated that the osteoporotic models were well established in the 8 months after OVX.

### Microstructural analysis of bone ingrowth

**Figure 5:**
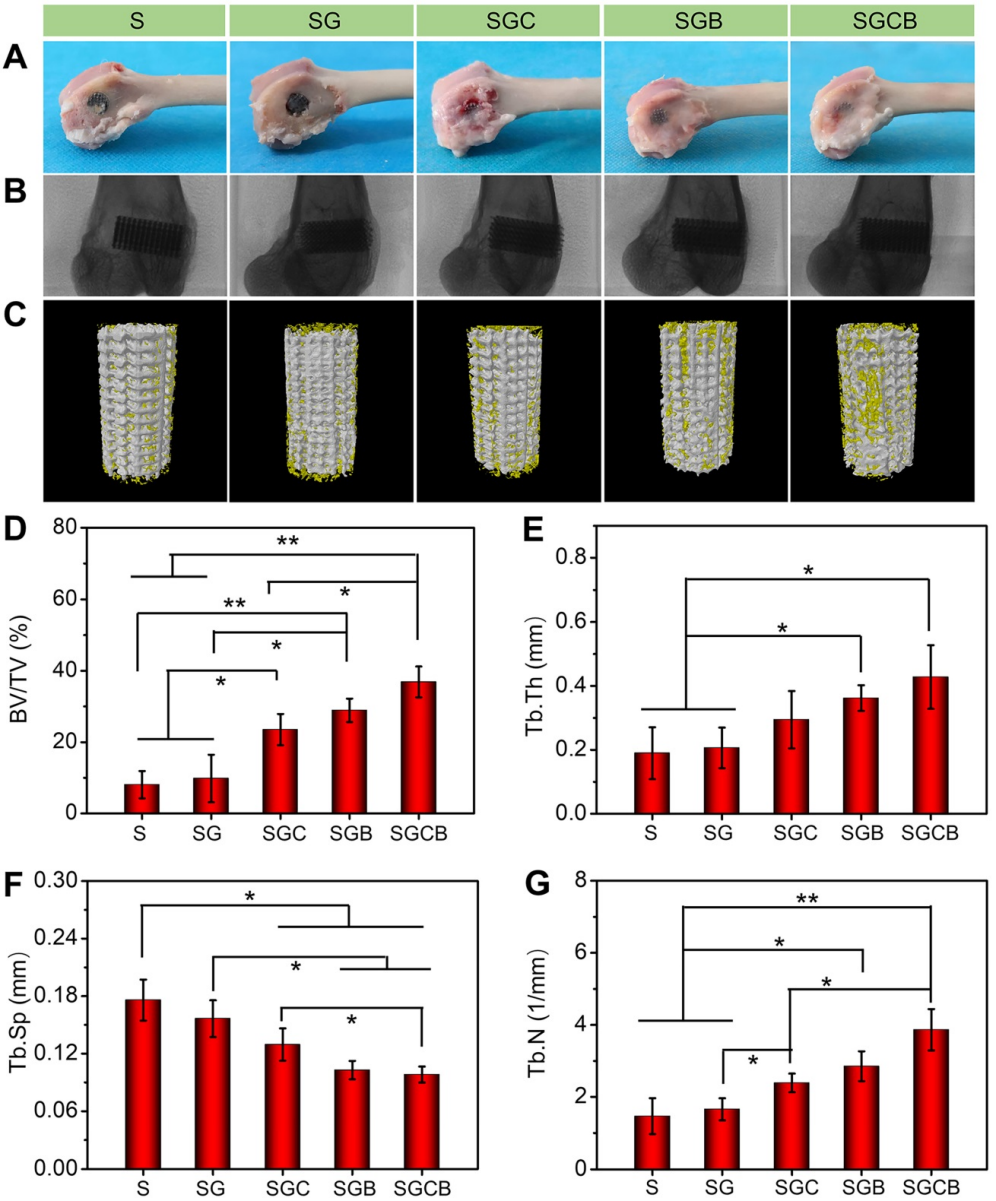
(**A**) Visual appearance and (**B**) radiographic images of the distal femurs; (**C**) 3D reconstruction images of ROI (bone was yellow and scaffolds were white); Quantitative analysis of (**D**) BV/TV, (**E**) Tb.Th, (**F**) Tb.Sp, and (**G**) Tb.N of the S, SG, SGC, SGB, and SGCB groups derived from micro-CT (*p < 0.05, **p < 0.01).

The main reason of limited bone regeneration in osteoporosis may be related to physiological defects of endogenous BMSCs and pathological osteoclast activity. Therefore, tissue engineering scaffolds are considered promising therapeutic systems owing to their combination of drug treatment and cellular therapy to improve interface osseointegration in osteoporotic bone defects 45. As shown in **Figure 5A**, visual appearance showed that all pTi scaffolds were fixed on the lateral condyle of the distal femurs. However, compared with the S and SG groups, those samples of the SGC, SGB, and SGCB groups showed the presence of some regenerated tissue on the scaffold surface. Radiographic images further proved that all the pTi were well fixed without evidence of loosening (**Figure 5B**). New bone formation was evaluated by Micro-CT in the porous titanium implant. The representative 3D reconstruction images were shown in **Figure 5C**, wherein it was clearly seen that more bone tissue (yellow) covered the surface and pore of scaffolds in the SGCB group, with moderate bone tissue coverage in the SGC and SGB groups, and limited bone tissue coverage in the S and SG groups. Statistical analyses of micro-CT were depicted in **Figure 5D-G**. The BV/TV values of S, SG, SGC, SGB, and SGCB groups were 8.1±3.8%, 9.8±6.7%, 23.5±4.3%, 28.9±3.3%, and 36.9±34.3%, respectively, which were consistent with 3D reconstruction images. The BV/TV value of the SGCB group was significantly enhanced compared with those of the S, SG (p < 0.01) and SGC (p < 0.05) groups, and slightly higher (p > 0.05) than that of the SGB group. The SGC and SGB groups also showed obvious differences with respect to the S and SG groups. In parallel, the Tb.N and Tb.Th of the SGC, SGB, and SGCB groups were higher than S and SG groups, and Tb.Sp of the SGC, SGB, and SGCB were lower than S and SG groups; and the SGCB group was the lowest of the five groups at 3 months.

### Osseointegration of bioactive interface with surrounding bone

**Figure 6:**
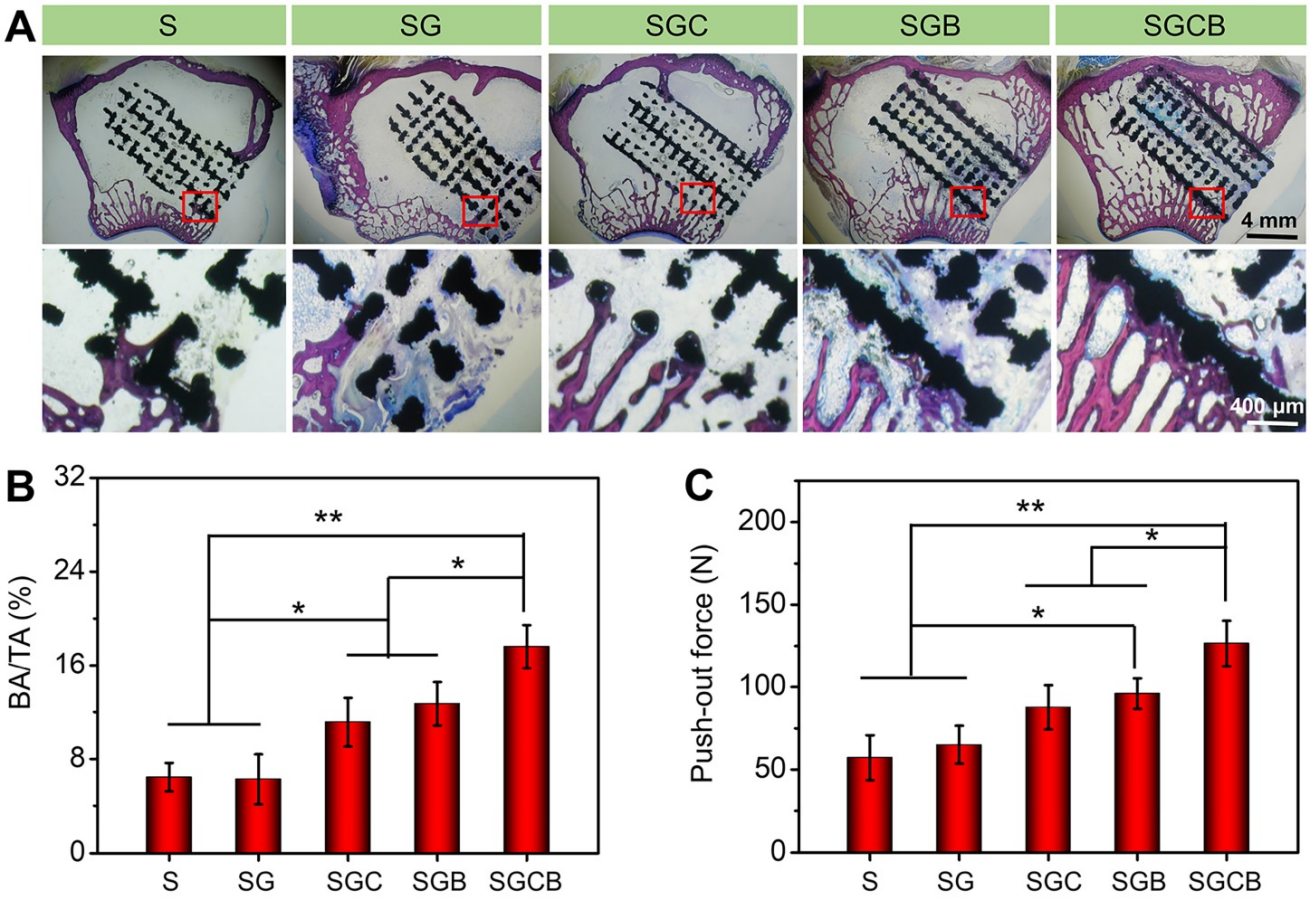
(**A**) Representative histological photos in the region of bone defect by Van-Gieson staining (The black areas stand for titanium alloys and the red areas stand for bones); (**B**) The ratio of regenerated bone area to total defect area (BA/TA) was analyzed using Image Pro Plus 6.0; (**C**) Evaluation of osseointegration through pull-out biomechanical testing after implantation for 3 months (*p < 0.05, **p < 0.01).

The histological results based on the Van-Gieson staining were applied to evaluate the bone ingrowth of different composition pTi scaffolds (**Figure 6**). As shown in** Figure 6A**, better bone regeneration was observed between the newly formed bone and pTi scaffold without gaps in the SGCB group, wherein thick regenerated bone almost completely surrounded the surface of the scaffold along with a part of bone tissue filling the inner pores. In addition, there was also massive new bone tissue concentrated at the peripheral pores of the SGB group, although not as significant as seen in the SGCB group. The bone regeneration was not obvious in the SGC group and showed gaps between the regenerated bone and scaffold. However, these results still demonstrated more new bone formation in the peripheral pores than those of S and SG groups, which had almost no bone ingrowth into the scaffold pores. To calculate the ratio of BA/TA, the histological images were analyzed by Image Pro Plus 6.0. As shown in **Figure 6B**, the BA/TA of SGCB group was significantly higher than SGC and SGB groups (p < 0.05), and S, SG groups (p < 0.01). There also were promoted areas of regenerated bone tissue in the SGC and SGB, when compared with the S and SG groups (p < 0.05).

To investigate the bone density and bone quality of distal bone, H&E and Masson's trichrome staining were performed on the bilateral proximal femurs (**Figure S8-11**). As shown in **Figure S8**, denser trabeculae were observed in the pTi scaffolds filled with active components (BMSCs or BMP-2 alone, or a combination) loaded hydrogel than in pTi scaffold with or without single hydrogel filling, as seen by a higher ratio of BA/TA on the left side (**Figure S9**). However, there was no difference in the trabecular structure on the right side (**Figure S10**). In the S and SG groups, the number of trabeculae on the right side was slightly higher than that on the left side, probably owing to the post-operative temporary disused osteoporosis of the left leg. Consistently, Masson's trichrome staining results revealed less intense red staining of the trabeculae on the left in the S and SG groups, which showed a degenerative change. While, no significant difference was observed in the right proximal femur (**Figure S11**). In summary, histological examinations of the left and right proximal femurs revealed a positive effect on the bone density and bone quality of trabeculae in the ipsilateral side after the implantation of the pTi scaffolds filled with BMSCs and/or BMP-2-loaded hydrogel, but no significant difference in the contralateral femurs.

### Mechanical properties of bioactive interface with surrounding bone

Given the superior bone regeneration, good osseointegration was formed between the pTi scaffolds and host bone. Hence, the push-out test was performed to further reveal the mechanical properties of bioactive interface with surrounding bone. The pull-out peak force values of the SGCB group were 2.2-fold and 1.95-fold higher than the S and SG groups (p < 0.01), and 1.5-fold and 1.3-fold higher than the SGC and SGB groups (p < 0.05) (**Figure 6C**), respectively. Incorporating BMP-2 in the composite scaffold also required a larger push force than that required with pTi with or without single hydrogel filling (p < 0.05). These results indicated that SGCB had the best result of interface bone integration.

Osteoporosis is a chronic disease and interface osseointegration after joint replacement in osteoporosis is a complex clinical problem. During this process, local transplantation of exogenous BMSCs with significant osteogenic activity could improve the pathological imbalance state of excessive osteoclast resorption and impaired osteogenic activity in osteoporosis, thus, to block the progression of osteoporosis 46, 47. BMSCs and BMP-2 are the common seed cells and growth factor for bone regeneration, respectively, and have been incorporated into scaffolds to enhance bone generation 48. Hydrogel, as a sustained release system, containing BMSCs and BMP-2 could effectively repair bone defects 49. However, there are limited studies on the use of hydrogels as cells or drug carriers to fill porous metal implants. In spite of BMSCs, exogenous BMP-2 and vascular endothelial growth factor were added into porous titanium scaffolds to promote long-term survival of titanium implants 50-52. However, these studies were investigated in non-osteoporotic models or only to unilaterally promote bone regeneration without comprehensive consideration of the mechanical strength of scaffolds, degradation rate of hydrogels, release profile, and the microenvironment of low osteogenic capacity in osteoporosis. Herein, the scaffolds with similar mechanical properties to bone and those hydrogels act as optimized BMSCs or/and BMP-2 delivery systems for enhancing bone regeneration significantly under osteoporotic conditions. Some severe complications, such as prosthesis interfacial displacement and loosening are is caused by the insufficient mechanical strength of the interface between prosthesis and bone tissue 53. Our push-out test result from different groups demonstrated that more stable bone-implant interface osseointegration were promoted by BMSCs and BMP-2 dual-loaded hydrogel, indicating significantly reduced risk of implant subsidence and displacement.

## Conclusion

Prosthetic interface osseointegration in osteoporosis is an urgent clinical problem, if improperly treated, which can cause serious complications, such as prosthesis subsidence, displacement and surrounding fracture. In this study, a novel 3D inorganic-organic bioactive interface, namely “3D printed metal microspores/ supramolecular hydrogel encapsulating BMSCs and BMP-2”, was constructed to targetedly ameliorate osteoporotic microenvironment. This composite system was demonstrated possessing good biocompatibility, ensured the sustained release of bioactive BMP-2, and was beneficial for osteogenic differentiation of BMSCs. As a synergic therapy, BMSCs and BMP-2 dual-loaded hydrogel could induce bone ingrowth and promote osseointegration of microporous titanium in osteoporotic bone defects. These findings suggested that this bioactive interface was a potentially promising candidate for the development of the artificial prosthesis interface for various osteogenesis- deficient patients, such as osteoporosis and rheumatoid arthritis.

## Supplementary Material

Supplementary figures and tables.

## Abbreviations

3D: three-dimensional
BMSCs: bone marrow mesenchymal stem cells
BMP-2: bone morphogenetic protein-2
pTi: porous titanium alloy
N-chitosan: N-carboxyethyl chitosan
ADH: adipic acid dihydrazide
HA-ALD: hyaluronic acid-aldehyde
DMEM: Dulbecco's Modified Eagle's Medium
FBS: fetal bovine serum
H&E: hematoxylin and eosin
CCK-8: Cell Counting Kit 8
PI: propidium iodide
DAPI: 4',6-diamidino-2-phenylindole
PBS: phosphate buffer saline
ALP: alkaline phosphatase
ELISA: enzyme-linked immunosorbent assay
micro-CT: microcomputed tomography
SEM: scanning electron microscope
CLSM: confocal laser scanning microscope
Con: nonporous Ti
S: pTi scaffold
SG: pTi scaffold filled with hydrogel
SGC: pTi scaffold filled with cell-loaded hydrogel
SGB: pTi filled with BMP-2-loaded hydrogel
SGCB: pTi filled with cells and BMP-2-loaded hydrogel
ARS: alizarin red staining
Runx-2: runt-related transcription factor-2
OCN: osteocalcin
Col-1: collagen type-1
OPN: osteopontin
RT-qPCR: real-time quantitative PCR
OVX: bilateral ovariotomy
ROI: region of interest
BV/TV: bone volume/tissue volume ratio
Tb.Th: trabecular thickness
Tb.Sp: trabecular separation
Tb.N: trabecular number
BMD: bone mineral density
BA/TA: the ratio of regenerated bone area to total defect area
