# Supplementary material for: Enhanced osseointegration of three-dimensional supramolecular bioactive interface through osteoporotic microenvironment regulation
Source: Theranostics. 2020 Mar 26;10(11):4779–94. doi: 10.7150/thno.43736 (PMC7163459; doi:10.7150/thno.43736)
Supplement: Supplementary file 1 — Supplementary figures and tables. [file thnov10p4779s1.pdf]

Supplementary data

Enhanced osseointegration of three-dimensional supramolecular bioactive interface through osteoporotic microenvironment regulation

Haotian Bai[1,3], Yue Zhao[2], Chenyu Wang[1,4], Zhonghan Wang[1,3], Jincheng Wang[1,3], Hou Liu[2], Yubin Feng[2], Quan Lin[2,✉], Zuhao Li[1,3,5,✉], He Liu[1,3,✉]

1. Orthopaedic Medical Center, The Second Hospital of Jilin University, Changchun 130041, P. R. China

2. State Key Lab of Supramolecular Structure and Materials, College of Chemistry, Jilin University, Changchun 130012, P. R. China

3. Orthopaedic Research Institute of Jilin Province, Changchun 130041, P. R. China

4. Department of Plastic and Reconstruct Surgery, The First Bethune Hospital of Jilin University, Changchun 130021, P. R. China

5. Department of Pain, Renji Hospital, South Campus, Shanghai Jiaotong University, Shanghai 201112, P. R. China

✉ Corresponding authors: He Liu (heliu@jlu.edu.cn), Zuhao Li (lizuhaojluedu@163.com), Quan Lin (linquan@jlu.edu.cn)

Haotian Bai and Yue Zhao contributed equally to this work.

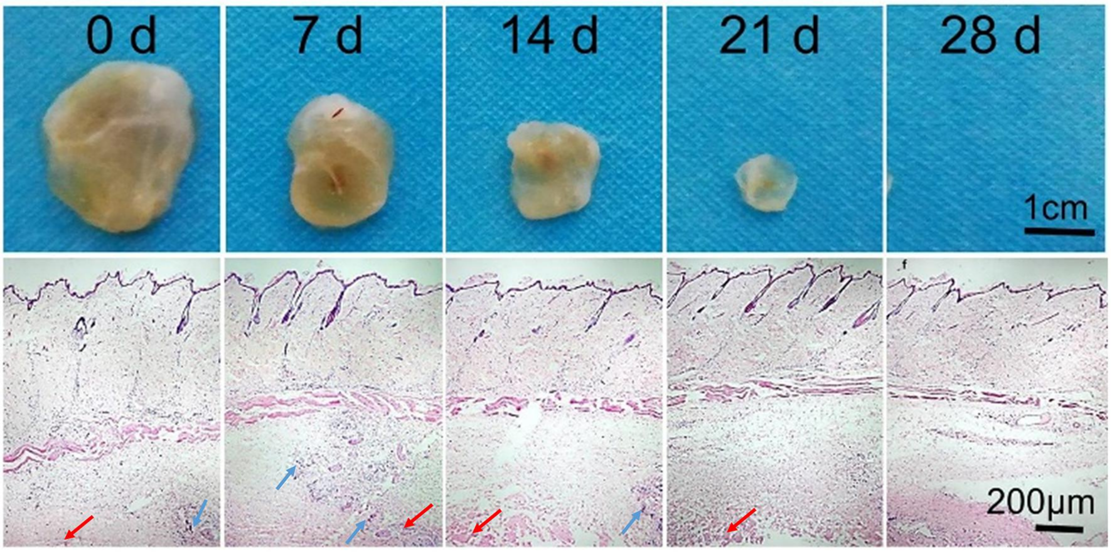

Figure S1. *In vivo* degradation and biocompatibility analysis of supramolecular hydrogel at different time points after subcutaneous implantation in rats. Blue arrows indicated the inflammatory cells and red arrows represented the hydrogel fragments.

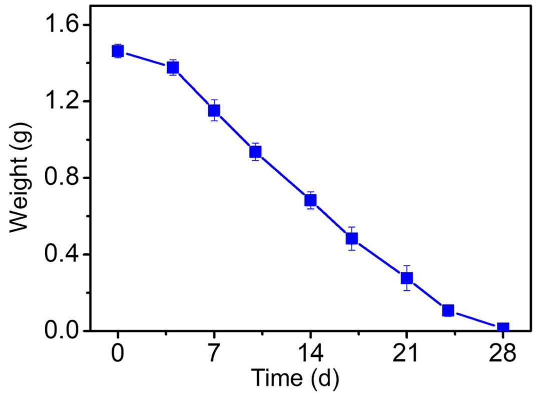

Figure S2. *In vivo* degradation of the supramolecular hydrogel by weighing.

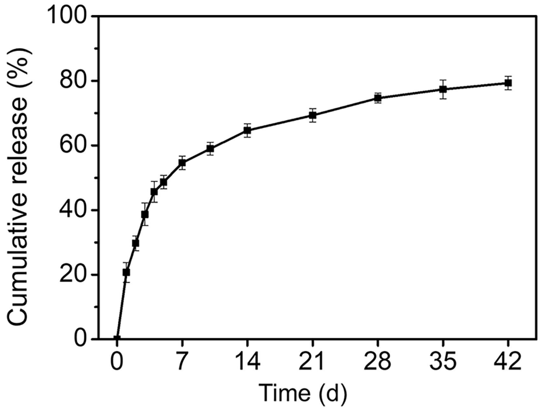

Figure S3. Release profiles of BMP-2 from the hydrogel/pTi constructs.

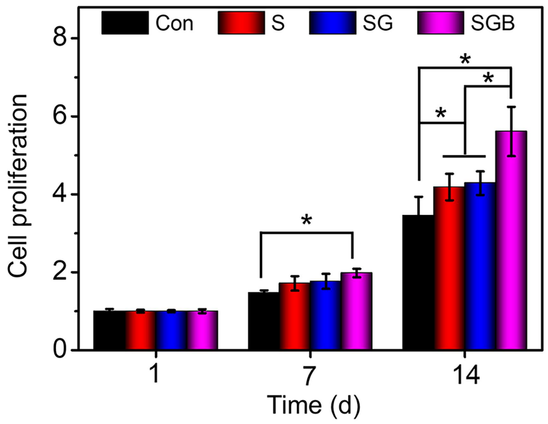

Figure S4. Cell proliferation on the different scaffolds for 1, 7, and 14 d (*p < 0.05).

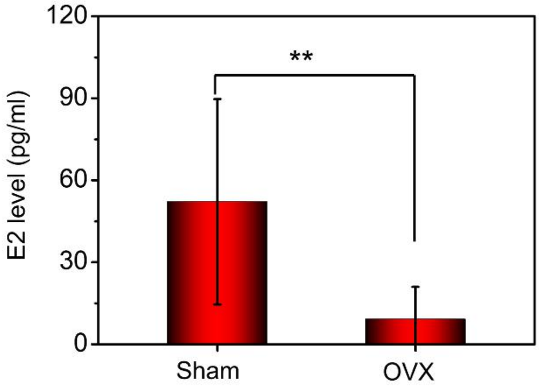

Figure S5. Serum estrogen (E2) levels of the OVX rabbit model 8 months after surgery (*p < 0.05).

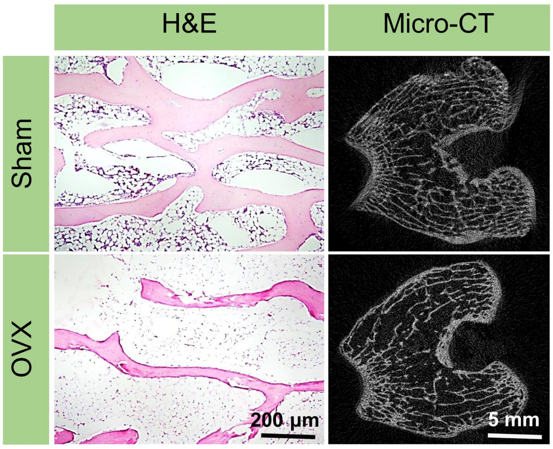

Figure S6. H&E staining (left, scale bar: 200 μm) and micro-CT slices (right, scale bar: 5 mm) of distal femurs show the trabecular bone structure of OVX rabbit model 8 months after surgery.

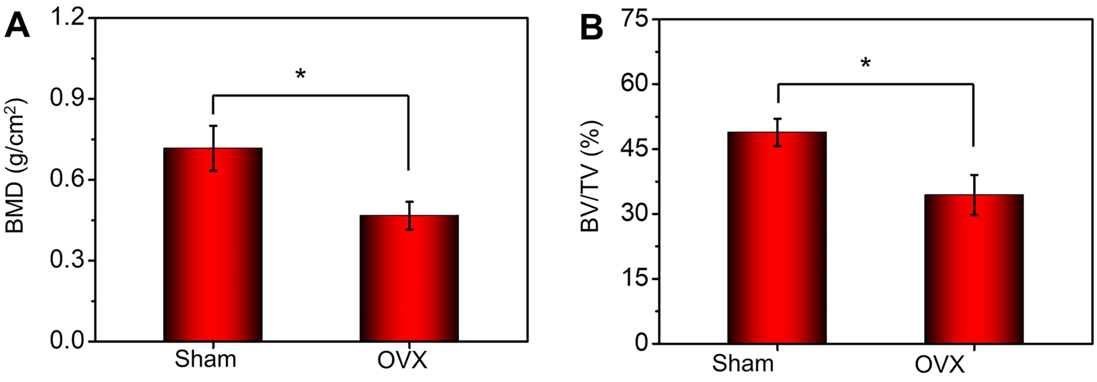

Figure S7. Quantitative analysis of (**A**) BMD and (**B**) BV/TV of OVX rabbit model 8 months after surgery by micro-CT images (*$p < 0.05$).

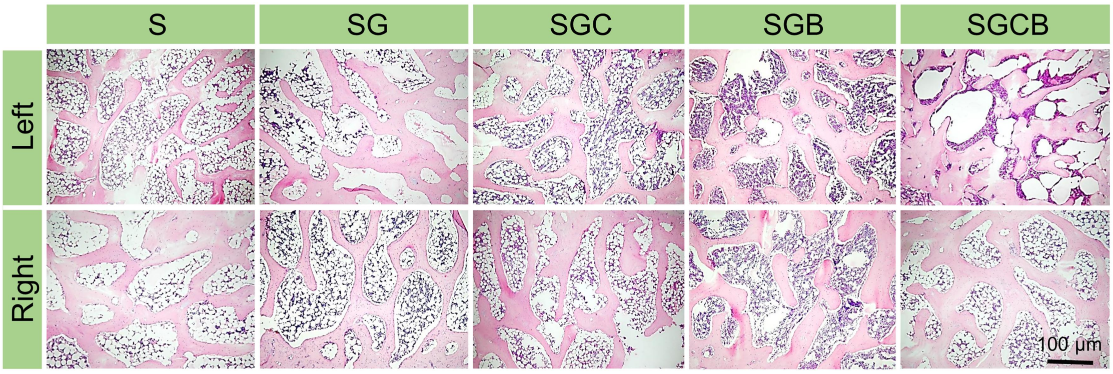

Figure S8. H&E staining of the effect on ipsilateral (left) and contralateral (right) proximal femurs 3 months after scaffold implantation.

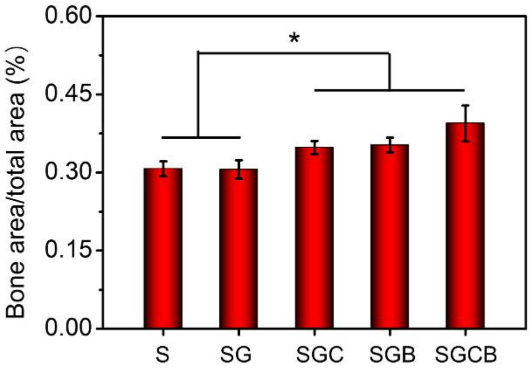

Figure S9. Quantitative analysis of ipsilateral (left) proximal femur bone area/total area (BA/TA) 3 months after scaffold implantation (*$p < 0.05$).

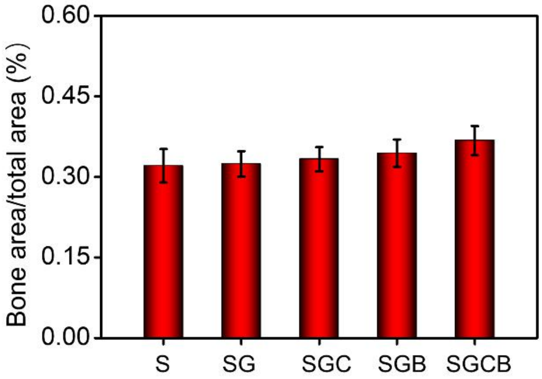

Figure S10. Quantitative analysis of contralateral (right) proximal femur bone area/total area (BA/TA) 3 months after scaffold implantation.

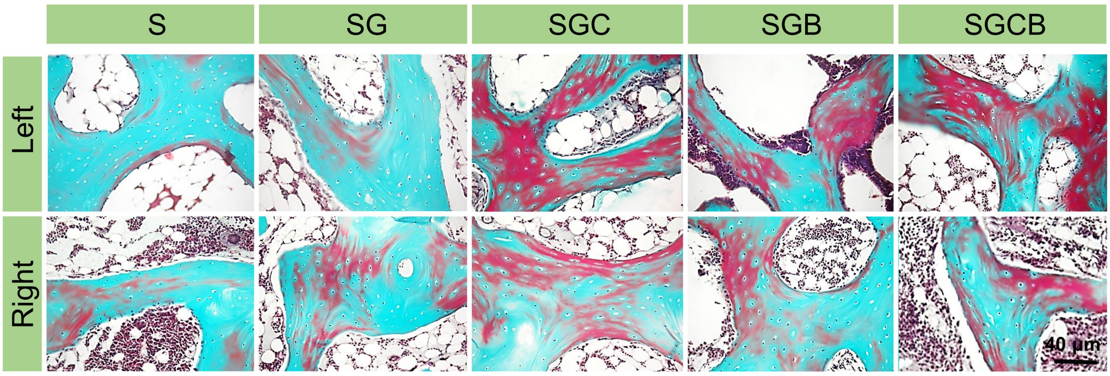

Figure S11. Masson's trichrome staining of the effect on ipsilateral (left) and contralateral (right) proximal femurs 3 months after scaffold implantation.

Table S1. The components of the pTi/BMP-2/BMSCs/supramolecular hydrogel constructs

Material	Components
BMP-2	Recombinant human BMP-2 (purity>95%)
BMSCs	Rabbit BMSCs
Supramolecular Hydrogel	N-Chitosan, ADH, HA-ALD
Ti6Al4V Scaffold	Ti (85.9%), Al (7.1%), V (5.2%)

Table S2. Primer sequences of genes

Gene subtype	Oligonucleotide Primers (5’-3’)
ALP	F: ATC GGA CCC TGC CTT ACC
	R: CTC TTG GGC TTG CTG TCG
Runx-2	F: ACTACCAGCCACCGAGACCA
	R: ACTGCTTGCAGCCTTAAATGACTCT
OCN	F: AGCCACCGAGACACCATGAGA
	R: AGCCACCGAGACACCATGAGA
Col-1	F: TCCGGCTCCTGCTCCTCTTA
	R: GGCCAGTGTCTCCCTTG
GAPDH	F: CAATGACCCCTTCATTGACC
	R: TGGACTCCACGACGTACTCA

Table S3. Characterizations of 3D printed pTi.

Characterization	Parameters
Porosity	
Archimedes'	70.5 ± 0.9 %
Micro-CT	69.2 ± 0.9 %
Pore size	793.4 ± 16.9 μm
Compressive strength	48.0 ± 2.1 MPa
Elastic modulus	1.63 ± 0.2 GPa
